# Cytogenetic analysis of three Ctenidae species (Araneae) from the Amazon

**DOI:** 10.1590/1678-4685-GMB-2020-0069

**Published:** 2020-11-16

**Authors:** José Paulo da Costa Pinto, Leonardo Gusso Goll, Maria Claudia Gross, Eliana Feldsberg, Carlos Henrique Schneider

**Affiliations:** 1 Instituto Nacional de Pesquisas da Amazônia – INPA Instituto Nacional de Pesquisas da Amazônia – INPA Programa de Pós-graduação em Genética Conservação e Biologia Evolutiva ManausAM Brazil Instituto Nacional de Pesquisas da Amazônia - INPA, Programa de Pós-graduação em Genética, Conservação e Biologia Evolutiva, Manaus, AM, Brazil.; 2 Instituto de Natureza e Cultura – INC Instituto de Natureza e Cultura – INC Benjamin ConstantAM Brazil Instituto de Natureza e Cultura – INC, Benjamin Constant, AM, Brazil; 3 Universidade Federal da Integração Latino-Americana Universidade Federal da Integração Latino-Americana Foz do IguaçuPR Brazil Universidade Federal da Integração Latino-Americana, Foz do Iguaçu, PR, Brazil.; 4 Centro Universitário União Dinâmica das Cataratas Centro Universitário União Dinâmica das Cataratas Foz do IguaçuPR Brazil Centro Universitário União Dinâmica das Cataratas, Foz do Iguaçu, PR, Brazil.

**Keywords:** Meiosis, FISH, NORs, spider, Amazon

## Abstract

Cytogenetic characterization was performed on three wandering spiders: *Ctenus amphora* Mello-Leitão, 1930, *C. crulsi* Mello-Leitão, 1930 and *C. villasboasi* Mello-Leitão, 1949. The three species had similar karyotypes, with 2n = 28 (26 + X_1_X_2_0) in males, with sex chromosomes exhibiting positive heteropicnosis in meiotic cells. 18S rDNA mapping revealed gene sites at the terminal region of one chromosomal pair for all species, with one *C. crulsi* individual, showing markings in two pairs. *C. villasboasi* showed markers only in the pachytene phase. The distribution pattern of constitutive heterochromatin was found to be characteristic for the genus, with markings in the centromeric region of all chromosomes, suggesting an acrocentric morphology for all chromosomes of the three analysed species. The results support the fusion of sex chromosomes as an evolutionary tendency for this spider group.

The order Araneae currently contains 48,455 described species, distributed in 120 families (World Spider Catalog, 2020). In Brazil, there are records of 3,203 species, of which 694 occur in the Amazon Region (Brescovit *et al.*, 2011). Within this group, we focus here on the Ctenidae family, a group which has received increasing attention due to their value as bioindicators of environmental quality, and the use of their venom neurotoxins as therapeutic agents (Rego *et al.*, 2005; Mestre and Gasnier, 2008; Pinheiro *et al.*, 2009).

More than 868 species of spiders have already been characterized cytogenetically and, of these, 12 belong to the family Ctenidae (Araujo *et al.*, 2020). The data for this family show a diploid number varying between 22 and 29 chromosomes for males, and X_1_X_2_0 and X_1_X_2_X_3_0 sex chromosome systems (Araujo *et al.*, 2014). While these two systems are rare overall, they are common in spiders (Araujo *et al.*, 2012), including the Ctenidae, making it possible, in this Family, to study the sex chromosome behavior during meiosis.

In Ctenidae, with the exception of *Asthenoctenus borellii* Simon, 1897, whose diploid number is 22, all species have 26 autosomal chromosomes plus two or three sex chromosomes (Araujo *et al.*, 2020). Araujo *et al.* (2014) suggest that the conversion of the sexual chromosomal system X_1_X_2_0 to X_1_X_2_X_3_0, and vice-versa, is a relatively common event. However, available cytogenetic data are currently insufficient to allow inferences concerning evolutionary chromosomal tendencies within the group.

The aim of the current study is to increase the cytogenetic knowledge of the Ctenidae, and so contribute to the discussion concerning mechanisms of chromosome evolution in this family, especially regarding the behavior of sexual chromosomal systems X_1_X_2_0 and X_1_X_2_X_3_0, in males during meiosis.

A total of 10 individuals (5 males of *Ctenus amphora* and 5 males of *C. crulsi*) were collected in a forest fragment surrounding the Federal University of Amazonas (UFAM), in the eastern part of the city of Manaus (03º04’34 “S, 59º57’30″ W), and 16 individuals (6 males of *C. amphora*, 8 males of *C. crulsi*, and 2 males of *C. villasboasi*) in the Adolpho Ducke Forest Reserve (2°57’42″S, 59°55’40″W) (Figure 1). In order to identify species of *Ctenus* we used Hofer *et al.* (1994). Collections were carried out under SISBIO license number 60728-1. Chromosomal preparation of male gonads was conducted according to Araujo *et al.* (2008). Fluorescent *in situ* hybridization (FISH) was performed following Pinkel *et al.* (1986), using 18S rDNA probes (Almeida *et al.*, 2010), that showed 91% homology with probes generated by Rincão *et al.* (2017) for *Ctenus ornatus*, and 92% homology for *Ctenus crulsi*. The sequences were compared in BLASTN, using the National Center for Biotechnology Information (NCBI) database website (https://blast.ncbi.nlm.nih.gov/Blast.cgi). For C-banding the Sumner (1972) protocol was followed. For Ag-NOR the Howell and Black (1980) protocol was followed.

**Figure 1:**
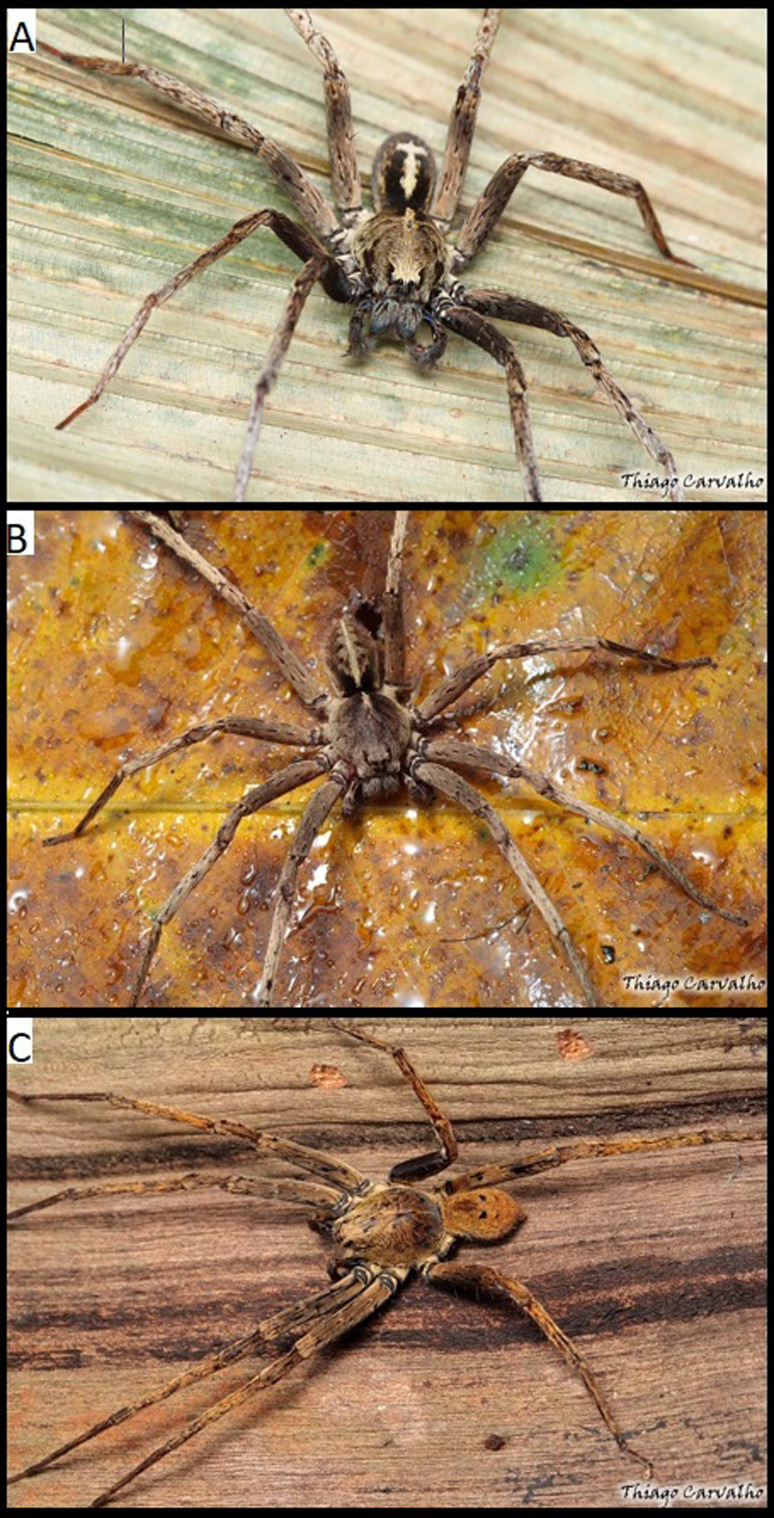
Spiders species analyzed in the present study: (A) Ctenus amphora and its specific orange spot in the form of an inverted amphore; (B) Ctenus crulsi showing four medium black spots on the abdomen with black antero-lateral borders; (C) Ctenus villasboasi easily recognized by the coloration and ventral white marks on the coxae and on the apex of the sternum. Photos were taken by Thiago Gomes de Carvalho.

In *C. amphora* (Figure 2A), *C. crulsi* (Figure 2B) and *C. villasboasi* (Figure 2C) 2n = 28, with 26 of the chromosomes being autosomal and two sexual. In pachytene (Figure 3A, D, G) and dipotene (Figure 3B, E, H) phase cells the sex chromosomes showed positive heteropicnosis, thus allowing their identification. Determination of the sex chromosomal system was performed by analyzing chromosome segregation during metaphase II of meiosis (Figure 3C, F, I), which shows nuclei with n = 13 and n = 15 (13 + X_1_X_2_).

**Figure 2:**
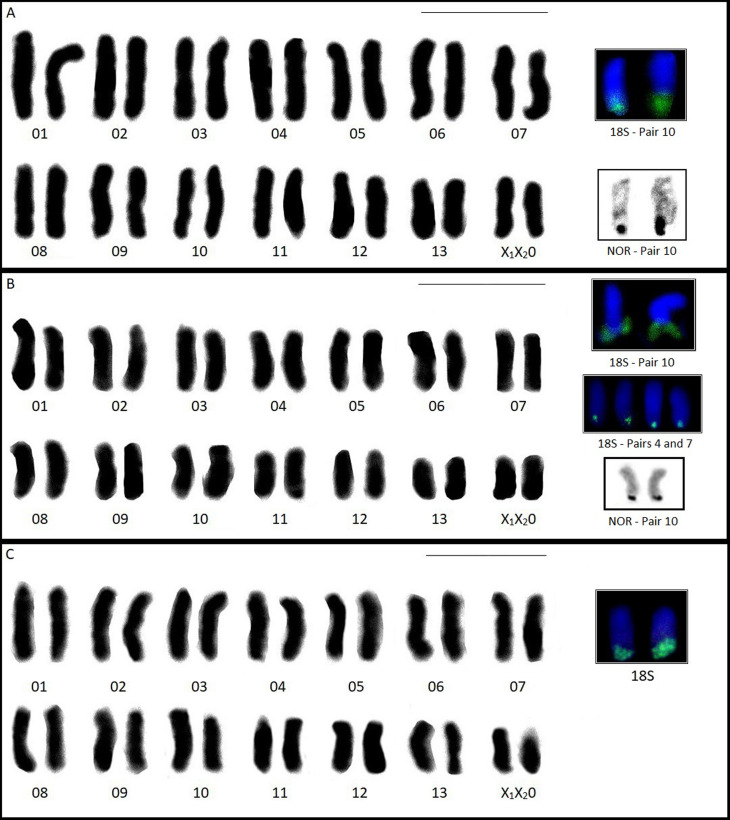
Karyotypes of the studied species: (A) Ctenus amphora; (B) C. crulsi and (C) C. villasboasi showing nucleolar pair marked by silver nitrate impregnation and FISH with 18S rDNA (Green marks). Scale= 10mm. One C. crulsi individual from the Ducke Reserve presented four labeled chromosomes (pairs 4 and 7) (Figure 1B - highlighted).

**Figure 3:**
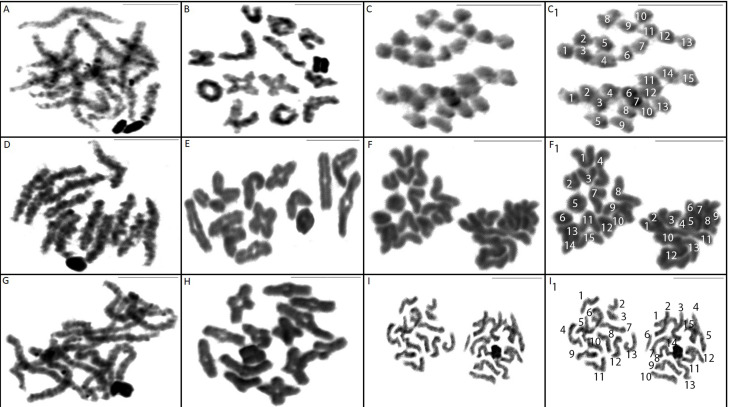
Meiotic cells in pachytene (A, D, G), diplotene (B, E, H) and metaphase II (C, F, I) of: Ctenus amphora. (A, B, C); C. crulsi. (D, E, F); C. villasboasi (G, H, I). Metaphase II cells shows the division of chromosome between cells poles, with n = 13 and n = 15 (13+X1X2) during segregation. Scale= 10mm.

The FISH technique revealed 18S rDNA sites on pair 10 of the chromosomes in the spermatogonial metaphase of *C. amphora* and *C. crulsi* (Figure 2A, B). In addition, a *C. crulsi* individual from the Ducke Reserve was polymorphic compared to the other seven analyzed for this same locus, with four labeled chromosomes (Pairs 4 and 7) in spermatogonial metaphases (Figure 2B - highlighted). *Ctenus villasboasi* showed marking on a pair of chromosomes in metaphase (Figure 2C). However, chromosomal size visualized with this technique was insufficient to allow determination of which pair carried the 18S rDNA site.

C-banding showed the presence of constitutive heterochromatin in the centromeric region of all chromosomes, characterizing the morphology as acrocentric for all three species (Figure 4A-C). Silver nitrate impregnation revealed NORs on pair 10 in the spermatogonial metaphases for *C. amphora* and *C. crulsi* (most individuals) (Figure 2A, B). *C. villasboasi* had no visible markings in the spermatogonial metaphases, but exhibited marking on one pachytene bivalent (Figure 4D).

**Figure 4:**
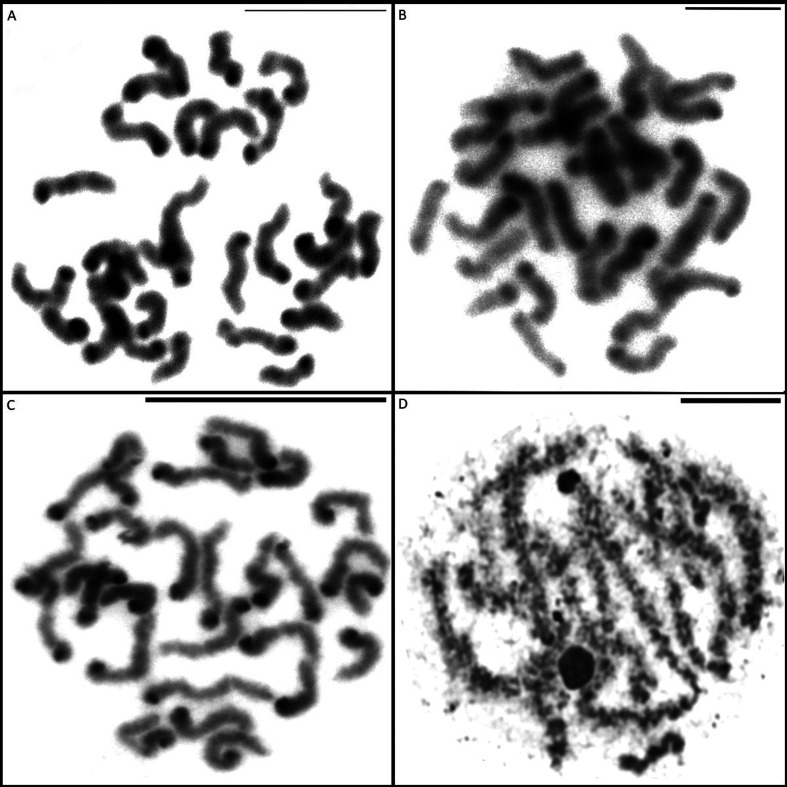
Mitotic cells submitted to C-banding (A, B e C) and silver nitrate impregnation (D). (A) C. amphora, (B) C. crulsi and (C) C. villasboasi showing heterochromatin in the centromeric region of chromosomes. (D) C. villasboasi showing NORs in pachytene bivalent. Scale= 10mm.

The karyotypic formula of males (2n=28 - 26+X_1_X_2_0), found for all species, has been shown to be conserved in *Ctenus* (Araujo *et al.*, 2020). Identification of sexual chromosomes in meiotic cells was facilitated by their high degree of condensation and positive heteropicnosis. The literature contains contrasting opinions regarding Ctenidae chromosomal morphology, with Araujo *et al.* (2014) considering them to be telocentric, while Kumar *et al.* (2017) and Rincão *et al.* (2017) consider that they are acrocentric. We considered the three species studied here to be acrocentric, based on C-banding, which showed markings in the centromeric regions of all chromosomes.

All three species showed metaphase II meiotic cells with n = 13 and n = 15 (13+X_1_X_2_), which agrees with the information given by Araujo *et al.* (2014) and Rincão *et al.* (2017) for Ctenidae species. This feature is common for a X_1_X_2_0 sex chromosomal system, in which for males, at the end of meiosis I, two sex chromosomes migrate to the same pole cell.

Araújo *et al.* (2012) collated proposed spider chromosomal evolution theories, which include the X0 sexual chromosomal system giving rise to the X_1_X_2_0 (White, 1940; Pätau, 1948), via part of an X chromosome fissioning and attaching to a supernumerary chromosome (Bole-Gowda, 1950), and the sexual chromosomal system X_1_X_2_X_3_0 giving rise to X_1_X_2_0 via fusion of two X chromosomes (Král, 2007).

Taking into account the cytogenetic characterization of species considered basal within the Ctenidae, such as *Nothroctenus* sp. and *Viracucha andicola* Simon, 1906 (Polotow and Brescovit, 2014), and the data presented here for Ctenidae species occuping positions considered to be derived, we believe the diploid number reduction hypotheses based on chromosome fusion to be more parsimonious, since the first group has 2n = 29 and the second group has 2n = 28.

However, Rincão *et al.* (2020) recently found two individuals of *C. ornatus* showing one supernumerary chromosome and one individual with two supernumerary chromosomes. Those chromosomes showed positive heteropicnosis and behavior similar to sex chromosomes, which the author states may demonstrate conversion of sexual chromosomal system X_1_X_2_0 to X_1_X_2_X_3_X_4_0 for the first time in Ctenidae.

The FISH-obtained 18S rDNA tags for the three species in our study confirmed Ag-NOR derived data, and were similar to those described for *C. ornatus* and *C. medius* by Rincão *et al.* (2017). However, an individual of *C. crulsi* in the current study had two pairs of chromosomes with 18S rDNA labeling (Pairs 4 and 7). These additional markers were shown to be relatively minor when compared to that found in the other *Ctenus* species of the current study, as well as those available in the literature. Such data suggest that these alterations can be caused by chromosomal rearrangements, insertions by transposable elements or ectopic recombination, all processes that could, potentially, be involved in karyotypic differentiation and new species emergence (Stáhlavsky *et al.*, 2018).

Considering the number of chromosomes with 18S rDNA in *C. crulsi*, we believe the translocation hypothesis to be the most feasible, since this mechanism of chromosome evolution depends on the interchange of segments between two non-homologous chromosomes without loss of genetic material (Gross *et al.*, 2010). Such translocations can be simple, when only the segment of one chromosome passes to the other, or reciprocal, when two chromosomes exchange segments with each other. The translocation model for rDNA is described by Araujo *et al.* (2015) for the exchange of such segments between autosomal and sexual chromosomes in the genus *Nephila*. This shows that a possible translocation between the two types of chromosomes is possible. In the *C. crulsi* metaphases analyzed here, the sex chromosomes were not evident.

Silver nitrate impregnation showed NOR markers on an autosomal pair for *C. amphora* and *C. crulsi* (most individuals), and on a pachytene bivalent for *C. villasboasi*, a result similar to that found for *C. ornatus* Keyserling, 1877 (Araujo *et al.*, 2014; Rincão *et al.*, 2020), and *C*. *medius* (Rincão *et al.*, 2020), but which differs from those reported for *C. indicus* Gravely, 1931, by Kumar *et al.* (2017), and for *G. longipes* by Rincão *et al.* (2020), who found NOR markings on two chromosome pairs. The NOR distribution pattern is currently known for only five species in the genus *Ctenus*. Therefore, we suggest that additional cytogenetic studies are still needed to establish the plesiomorphic characteristics of the Ctenidae karyotype and, thus, to be able to understand the mechanisms of chromosomal evolution that occurred in this group of spiders.

The pattern of constitutive heterochromatin distribution reported here for *C. amphora, C. crulsi* and *C. villasboasi* is similar to those found by Rincão *et al.* (2017) for *C. medius* Keyserling, 1891, *E. cyclothorax* Bertkau, 1880, *P. nigriventer* Keyserling, 1891 and *V. andicola*, with blocks in the centromeric regions of all the chromosomes.

According to Sumner (1972), C-banding marks centromeric and telomeric regions, possibly marking nucleolar regions and, rarely, intercalated regions of the chromosome. In the metaphases studied here, the sex chromosomes were not totally heterochromatic, thus confirming heteropycnosis of the sex chromosomes in meiosis. Heterochromatic chromosomes remain condensed throughout the cell cycle, whereas heteropycnotic chromosomes may have a higher or lower level of condensation, depending on the stage of cell division in which they are found (John, 1990).

As a result of the current study, the number of Ctenidae species with chromosomal data has been increased to 14. The data obtained for *C. amphora, C. crulsi* and *C. villasboasi* extends to seven the number of species analyzed from the genus *Ctenus*, and so allow a conserved karyotype to be infered, as well as the diploid number, chromosomal formula and sex chromosome system plus mapping of the rDNA sites, constitutive of sex chromosomes in meiotic cells in pachytene and diplotene phases is a common feature for these species (Araújo *et al.*, 2012). The results also reinforce theories of chromosome fusion as a possible evolutionary tendency in this family.
